# Conductometric H_2_S Sensors Based on TiO_2_ Nanoparticles

**DOI:** 10.3390/ma17133283

**Published:** 2024-07-03

**Authors:** Yassine Alaya, Malek Madani, Noureddine Bouguila, Lassaad El Mir, Enza Fazio, Carmelo Corsaro, Giovanni Neri

**Affiliations:** 1 Laboratory of Physics of Materials and Nanomaterials Applied at Environment (LaPhyMNE), Faculty of Sciences in Gabes, Gabes University, Gabes 6072, Tunisia; yassine1alaya@gmail.com (Y.A.); malek.madani@univgb.rnu.tn (M.M.); bouguila.nour@gmail.com (N.B.); lassaad.elmir@fsg.rnu.tn (L.E.M.); 2 Department of Mathematical and Computer Sciences, Physical Sciences and Earth Sciences, University of Messina, Viale F. Stagno D’Alcontres 31, 98166 Messina, Italy; enza.fazio@unime.it (E.F.); carmelo.corsaro@unime.it (C.C.); 3 Department of Engineering, University of Messina, C.da Di Dio, 98166 Messina, Italy

**Keywords:** sol-gel, TiO_2_ nanopowder, thermal treatment, H_2_S sensor

## Abstract

High-performance hydrogen sulfide (H_2_S) sensors are mandatory for many industrial applications. However, the development of H_2_S sensors still remains a challenge for researchers. In this work, we report the study of a TiO_2_-based conductometric sensor for H_2_S monitoring at low concentrations. TiO_2_ samples were first synthesized using the sol-gel route, annealed at different temperatures (400 and 600 °C), and thoroughly characterized to evaluate their morphological and microstructural properties. Scanning electronic microscopy, Raman scattering, X-ray diffraction, and FTIR spectroscopy have demonstrated the formation of clusters of pure anatase in the TiO_2_ phase. Increasing the calcination temperature to 600 °C enhanced TiO_2_ crystallinity and particle size (from 11 nm to 51 nm), accompanied by the transition to the rutile phase and a slight decrease in band gap (3.31 eV for 400 °C to 3.26 eV for 600 °C). Sensing tests demonstrate that TiO_2_ annealed at 400 °C displays good performances (sensor response Ra/Rg of ~3.3 at 2.5 ppm and fast response/recovery of 8 and 23 s, respectively) for the detection of H_2_S at low concentrations in air.

## 1. Introduction

With the swift evolution of the global industry and the desire to improve air quality, hydrogen sulfide (H_2_S) has been recognized as one of the highly concerned pollution gases, commonly emitted by industries operating in the fields of pulp and paper manufacturing, natural gas, biological decomposition of organic waste material, and crude petroleum [1,2,3]. H_2_S is a hazardous chemical, colorless, and extremely flammable [4,5]. At low concentrations, it has an odor of rotten egg, which may cause coughing and sore throat and eyes, while people exposed to high concentrations (300–500 ppm) may experience the human olfactory nerve system and the collapse of the cardiovascular system [6]. Therefore, it is mandatory to develop H_2_S sensors with good performance.

Among the variety of sensors used for gas sensing, conductometric sensors have proven to be pretty attractive for detecting a variety of gases, since they are easy to fabricate, low cost, and simple to operate [7,8,9]. In the literature, there are many reports on H_2_S sensors based on metal oxide semiconductors (MOS), such as Fe_2_O_3_ [10], CuO [11], ZnO [12], WO_3_ [13], and NiO [14]. However, TiO_2_-based H_2_S sensor development is still scarce, and their gas-sensing performance needs to be improved [15]. TiO_2_ has intriguing physical and chemical features, making it a promising choice for gas sensor applications due to its distinct allotropic phases (anatase, rutile, and brookite) [6]. This involves the microstructural, morphological, and defect characteristics, which can play a crucial role in enhancing sensor response. Meanwhile, selecting synthesis techniques for the TiO_2_ nanoparticles is a vital step to achieving a larger surface area with higher roughness. Various physical and chemical routes are commonly used for the synthesis of TiO_2_ nanoparticles, such as Pulsed Laser Deposition (PLD) [16], sol-gel [17], thermal evaporation [18], sputtering [15], spray pyrolysis [19], and Atomic Layer Deposition (ALD) [20]. TiO_2_ has a high surface area, enhancing its interaction with gas molecules and improving sensitivity [21,22,23]. It is chemically stable and corrosion-resistant, ensuring long-term durability and reliability. TiO_2_ exhibits excellent photocatalytic activity [24], significantly changing its conductivity when exposed to light and gas molecules, which enhances sensitivity and response time. The material can be synthesized in various nanostructured forms, providing greater surface area and more active sites for gas adsorption. Additionally, TiO_2_ is cost-effective and abundantly available, making it an economical choice for gas sensor development. Therefore, these features provide the TiO_2_-based sensor with great sensitivity and selectivity for hydrogen sulfide.

In this study, we have synthesized TiO_2_ nanoparticles (NPs) with the modified sol-gel method using ethyl alcohol under supercritical conditions, which requires lower energy consumption and allows the synthesis of materials with high purity and homogeneity. We investigated their structural, morphological, and optical properties and their performances in gas-sensing for detecting low hydrogen sulfide concentrations in the range from 0.5 to 4 ppm. The developed sensor exhibited enhanced sensitivity, selectivity, and fast response/recovery times to H_2_S.

## 2. Experimental Section

### 2.1. Synthesis of TiO_2_ Nanopowder

**Figure 1:**
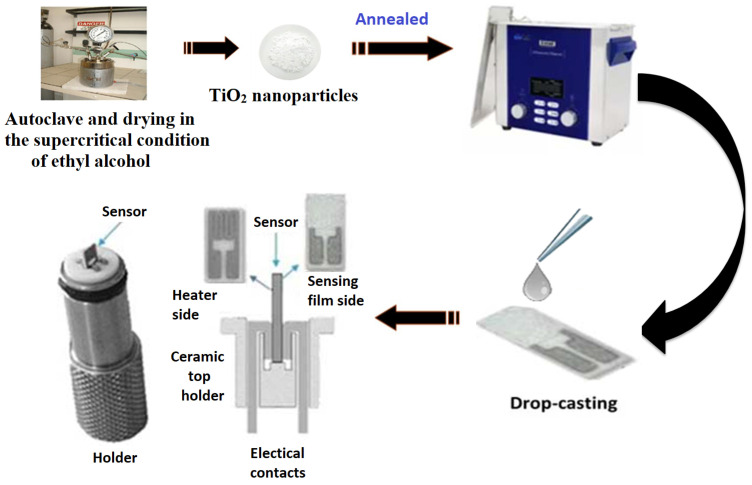
Fabrication process of the TiO_2_ conductometric sensor.

TiO_2_ nanopowder was prepared using the protocol of El Mir et al. [25,26] based on the following steps. First, 15 mL of Titanium (IV) isopropoxide Ti(OC_3_H_7_)_4_ (97%, from Sigma–Aldrich, Saint Louis, MO, USA) was dissolved in 45 mL of methanol blended with 2 mL of acetic acid (CH_3_COOH). The mixture was kept under magnetic stirring until the precursors were completely dissolved. The resulting solution was then poured into the autoclave to achieve drying in supercritical conditions of 250 mL of ethanol (Tc = 243 °C; Pc = 63.6 bars), with a heating rate of 45° C/h. Afterward, the as-obtained nanopowder was calcined for 2 h in air at different temperatures, (T = 400 °C) and (T = 600 °C). For the preparation of the TiO_2_ conductometric sensor, a quantity of 1 mg of TiO_2_ powder was sonicated for 30 min with 1 mL of deionized water. The gas sensor was manufactured in the temperature range between 20 °C and 25 °C. A scheme of the synthesis procedure is illustrated in Figure 1.

### 2.2. Characterizations

Microstructural analysis was determined with a D2 phaser Bruker X-ray diffractometer (Bruker, Billerica, MA, USA) using the Cu Kα line (0.159 nm) in the 10–80° 2θ range. FT-IR spectra were recorded utilizing a PerkinElmer spectrometer (PerkinElmer, Waltham, MA, USA) equipped with a universal attenuated total reflectance (ATR) sampling accessory. The UV–visible diffuse reflectance spectra (UV–visible DRS) were measured using a Shimadzu (Kyoto City, Japan) 2600–2700 spectrometer with BaSO_4_ as a reference. Raman spectra of the samples were recorded using the XploRa Raman spectrometer (Horiba Scientific, Piscataway, NJ, USA) equipped with an Olympus BX-40 microscope (Olympus, Tokyo, Japan) (objective ×50 focal length), Peltier cooled CCD detector, 532 nm diode laser, and 600 L/mm grating. The laser power was 5 mW, and the acquisition time was 30 s. Two to ten spectra were registered for each sample at different positions to verify sample homogeneity. The reference spectrum of Si (peak position of 521 cm^−1^) was measured to avoid temperature drift. Scanning Electron Microscope (SEM) images were taken using a Zeiss (Oberkochen, Germany) (Gemini II) microscope at the acceleration voltage of 5 kV.

### 2.3. Gas Sensing Tests

The gas sensing tests were carried out with sensors fabricated by printing TiO_2_ on the sensor device with a heating element and Pt-interdigitated electrodes. For the tests, the sensor devices were introduced into the test chamber. An Agilent E3632A instrument (Agilent, Santa Clara, CA, USA) was employed for setting the operating temperatures, whereas the resistance of the TiO_2_ sensing layer was measured with an Agilent 34970A multimeter (Agilent, Santa Clara, CA, USA). H_2_S sensing tests were carried out under a flow of dry synthetic air of 100 cc/min, operating at temperatures from 100 to 400 °C, with H_2_S gas concentrations of 0 to 4 ppm. The gas response, S, is defined as the ratio R_a_/R_g_ for n-type behavior, where R_a_ is the baseline resistance in dry synthetic air and R_g_ is the electrical resistance at different gas concentrations. The response time, τ_res_, and recovery time, τ_rec_, were defined as follows. Response time, τ_res_, i.e., the time required for the sensor to reach 90% of the saturation resistance after injection of the target gas, and recovery time, τ_rec_, i.e., the time required for the sensor to reach 90% of the resistance baseline value in air. These were also evaluated.

## 3. Results and Discussion

### 3.1. Sample Characterizations

**Figure 2:**
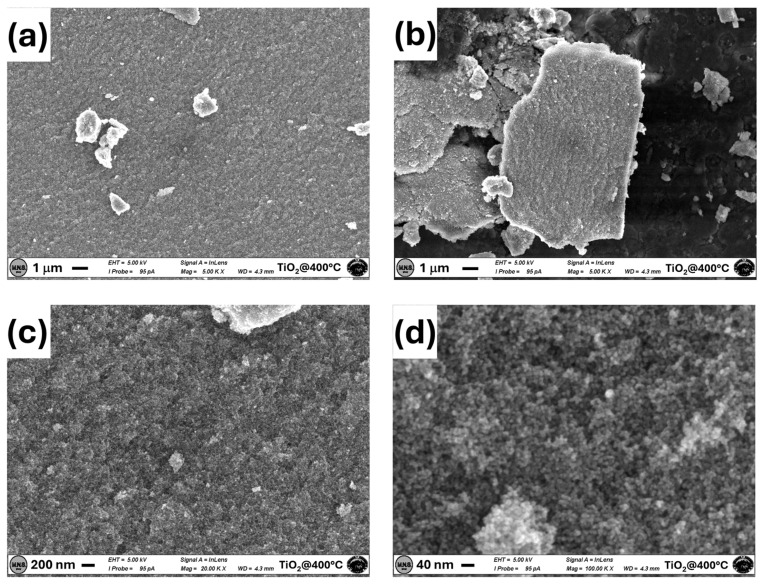
SEM images of the TiO_2_ sample annealed at 400 °C acquired at 5k magnifications in two different regions. (**a**,**b**) At 20k magnifications (**c**) and 100k magnifications (**d**).

TiO_2_ samples synthesized using the sol-gel route and annealed at different temperatures (400 and 600 °C) were first thoroughly analyzed by different characterization techniques. The SEM images of the TiO_2_ sample annealed at 400 °C are reported in Figure 2.

The sample annealed at 400 °C is not completely homogeneous, showing regions with different microscopic features characterized by randomly distributed and non-uniform clusters of TiO_2_ (Figure 2a,b). However, all regions show a high porosity on a nanometric scale with grain size in the range of 10–20 nm (Figure 2c,d).

**Figure 3:**
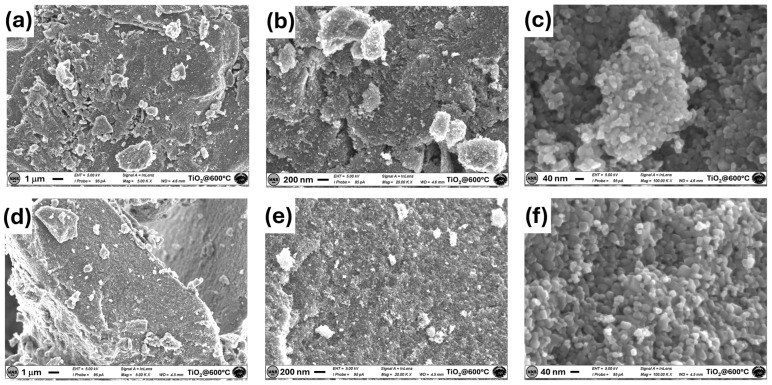
SEM images of the TiO_2_ sample annealed at 600 °C were acquired in two different regions at 5k magnifications (**a**,**d**), 20k magnifications (**b**,**e**), and 100k magnifications (**c**,**f**).

Figure 3 reports characteristic SEM images of the TiO_2_ sample annealed at 600 °C. The morphology of this sample is more homogeneous, with a bigger grain size in the 30–50 nm range and a fractal-like structure induced by calcination. In addition, it can be noted that, at the highest annealing temperature, the collapse of the mesostructure occurs, which could be caused by the crystallization of the amorphous titania into nanosized anatase particles and/or with the transition from the anatase to the rutile phase.

**Figure 4:**
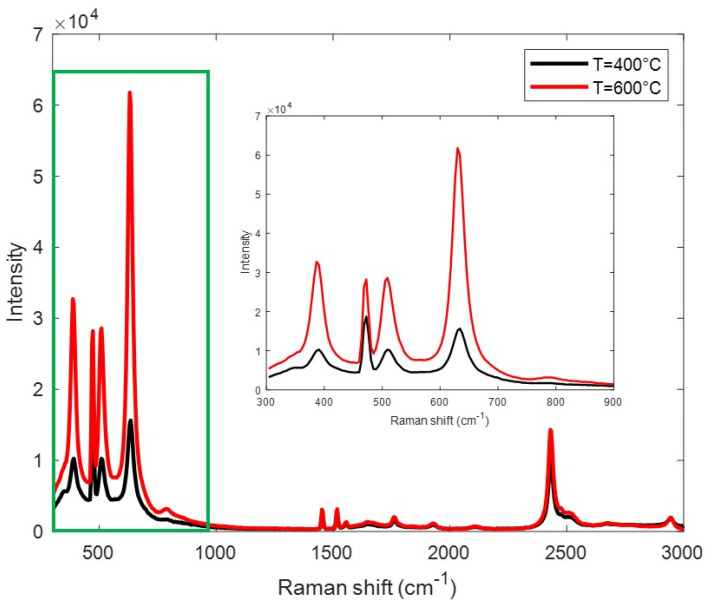
Raman spectra of the samples annealed at 400 °C and 600 °C. The inset shows an enlargement of the spectral region between 300 and 900 cm^−1^.

To investigate this transition phase process, further characterizations have been carried out. The vibrational properties were investigated by Raman measurements, the profiles of which are reported in Figure 4. The three Raman peaks centered at about 386, 509.3, and 630.3 cm^−1^ (inset in Figure 4) are assigned to the Raman active modes of the anatase TiO_2_ crystalline structure, while the peak at about 472.5 cm^−1^ is associated with the Raman active modes of the rutile crystalline phase [27]. The bands in the region higher than 1500 cm^−1^ are due to C-C and C-H/C-O contributions, which is in good agreement with FTIR data. Ultimately, Raman evidence indicates that the two TiO_2_ phases characterize the investigated samples. However, the relative intensity of the peaks of the anatase phase compared to the peak associated with the rutile phase is different in the two samples. This indicates that the phase transition from the anatase phase to the rutile phase occurs at the highest temperature.

**Figure 5:**
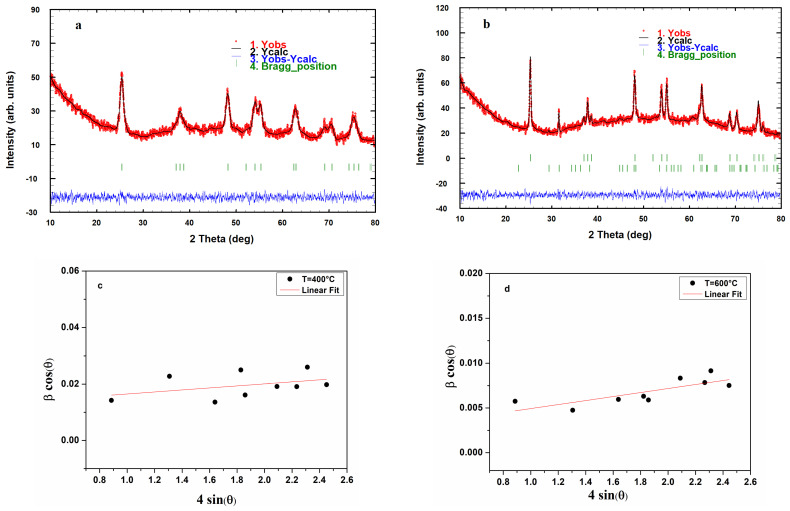
(**a**,**b**) Rietveld refinement of the X-ray diffraction profile and (**c**,**d**) Williamson–Hall plots of TiO_2_ nanopowders calcined at 400 °C and 600 °C.

In Figure 5, the XRD patterns of both TiO_2_ samples are shown. In Figure 5a, which shows the XRD pattern of TiO_2_ powder annealed at 400 °C, we identified the (101), (103), (004), (112), (200), (105), (211), (204), (116), (220), and (215) diffraction peaks ascribed to the TiO_2_ tetragonal structure in the anatase phase (JCPDS 21-1272) [28,29]. As shown in Figure 5b, upon increasing the annealing temperature to 600 °C, the diffraction peaks are narrower and slightly more intense. We can also discern the orthorhombic structure of TiO_2_ as discerned by its characteristic (011) peak centered at 31.7° (JCPDS 80-5176) [30].

The Rietveld refinements of the crystal structures of the as-prepared TiO_2_ samples were carried out using the FullProf software (https://www.ill.eu/sites/fullprof/, accessed on 6 May 2024). The method employs a least-squares procedure to compare Bragg intensities and those calculated from a possible structural model. In the first step of refinement, the global parameters, such as background and scale factors, were refined. In the next step, structural property parameters such as lattice parameters, profile shape and width parameters, preferred orientation, asymmetry, isothermal parameters, atomic coordinates, and site occupancies were refined in sequence.

The average crystallite size and the lattice strain were calculated according to the Williamson–Hall method using the following equation [31]:

(1) $$\beta \cos\theta =\frac{K\lambda }{D}+4\varepsilon \sin\theta$$

where β is the peak full width at half maximum (FWHM), θ is the Bragg angle, *K* is the shape factor (0.9), λ is the incident wavelength (λ = 1.5406 Å), and ε is the film strain. The trend of βcosθ as a function of 4sinθ for the investigated samples is shown in Figure 5c,d.

The fitting quality of the experimental data is assessed by computing parameters such as the ‘goodness of fit’ χ^2^, the Bragg R-factor, and the Rf-factors (Profile R-factor (Rp), Weighted Profile R-factor (Rwp), and Expected R-factor (Rexp)). The values of these structural parameters are reported in Table 1.

The results deduced from the Rietveld refinements of the XRD profiles are reported in Table 2, giving information about the significant variation of the phase composition and the crystallite size. It emerges that the crystallite size of the TiO_2_ sample increases upon increasing the annealing temperature.

**Figure 6:**
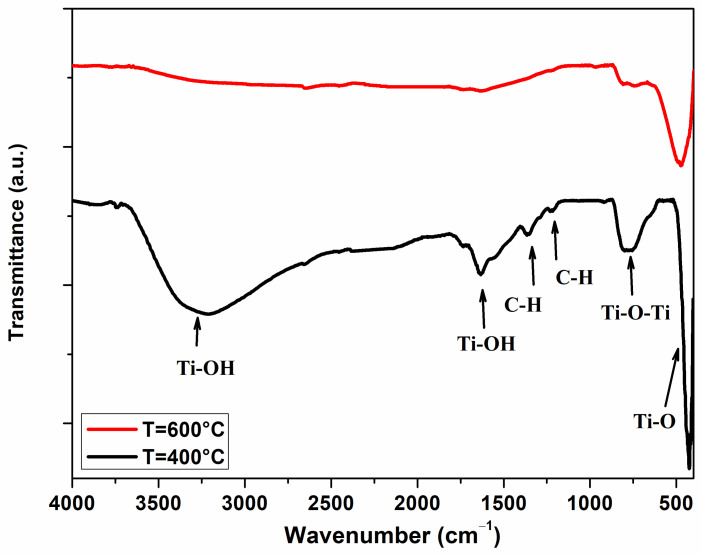
FTIR spectrum of TiO_2_ nanopowder annealed at 400 °C and 600 °C.

Figure 6 shows the FTIR spectra of TiO_2_ samples in the 400–4000 cm^−1^ range. The spectrum of the sample annealed at 400 °C shows two broad bands, centered at about 500 and 860 cm^−1^, which are assigned to the Ti–O bending and Ti−O−Ti stretching vibrations [32], respectively. Furthermore, the barely visible contributions at about 1240 and 1340 cm^−1^ are ascribed to the C-H twisting and bending vibrational modes [33,34], whereas the two bands at around 1630 and 3310 cm^−1^ correspond to the presence of related hydroxyl groups (Ti-OH) and those of water molecules [35,36]. As expected, when calcination temperature increases, peaks relative to the hydroxyl groups and adsorbed C-H disappear. At the same time, we observed a slight change in the diffraction profile of TiO_2_, indicating the rearrangement of the Ti-O network to facilitate the crystallization of TiO_2_ [32].

This agrees with what is known from the literature [37], which reports that although the oxygen content remains constant up to annealing temperatures of 900 °C, when there is an increase in temperature, there is also an increase in “O^−^ species” due to the hydroxyl groups and carbon impurities desorbing from the surface. This process, due to the localized charge transfer between anionic and cationic frameworks during thermally induced reduction, favors the sensing mechanism of H_2_S, which reacts with the adsorbed oxygen species to form SO_2_ and H_2_O (see Section 3.3).

**Figure 7:**
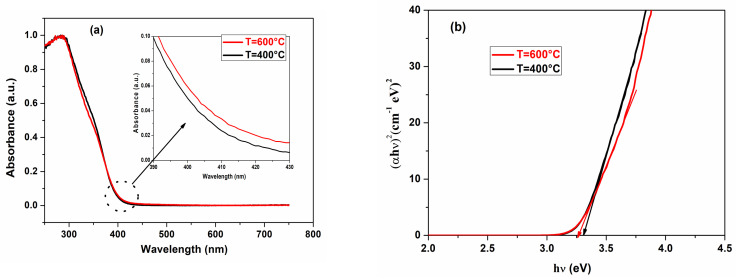
Absorbance spectra (inset shows the observed shift) (**a**) and Tauc plots (**b**) of TiO_2_ nanopowders prepared using the sol-gel technique and annealed at different temperatures.

UV–visible absorption measurements were carried out to investigate the changes in optical transitions of TiO_2_ nanostructures caused by annealing. The Kubelka–Munk equation was used to calculate the absorption spectra of the samples from the diffuse reflectance spectra [26]. Figure 7a shows absorbance spectra with wavelengths ranging from 250 to 800 nm for both samples synthesized using the sol-gel method. The absorbance of the nanostructures is around 90% in the UV range and decreases dramatically beginning in the visible range. Seemingly, annealing has no significant impact on the absorbance of the ceramic in the UV range. The bandgap energy E_g_ of TiO_2_ nanostructures was estimated according to the Tauc method, following Equation (2) [29,38,39]:

(2) $${\left(\alpha h\nu \right)}^{2}=A\left(h\nu -\mathrm{Eg}\right)$$

In this equation, α is the absorption coefficient, A is a constant, and hν is the photon energy. Extrapolating the linear part of the curve to the hν-axis yielded the optical band gap, as illustrated in Figure 7b. The estimated band gap energy values were 3.31 eV and 3.26 eV for samples annealed at 400 °C and 600 °C, respectively. Similar behavior was reported in the literature for TiO_2_ nanostructures synthesized using the sol-gel method [29].

### 3.2. Gas Sensing Tests

**Figure 8:**
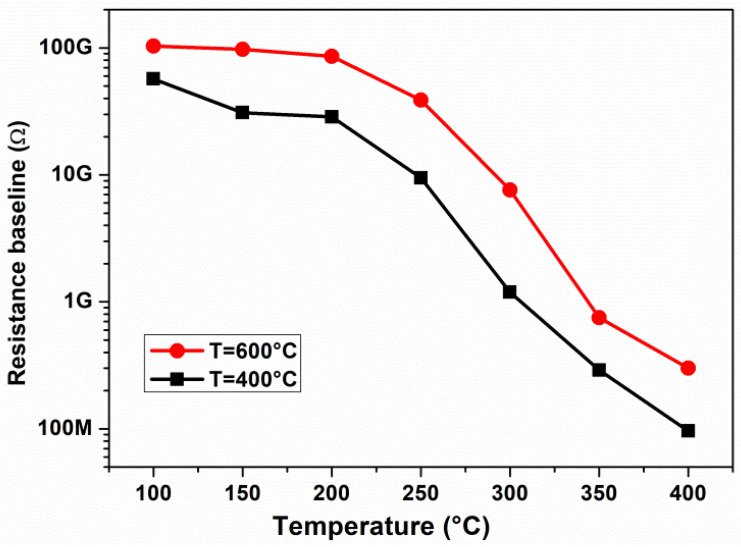
Baseline resistance of TiO_2_-NP sensors vs. operating temperature.

Before investigating the gas sensing properties, the baseline resistance of the TiO_2_ layer, denoted as R_a_, versus operating temperature has been investigated (see Figure 8). The sensor baseline displays a higher resistance at low temperatures. As the temperature increases, the resistance baseline decreases because of the thermal excitation of electrons into the conduction band, indicating the semiconductor behavior of TiO_2_. The data have further shown that TiO_2_ (600 °C) is more resistive, due to the presence of the rutile phase, compared to TiO_2_ (400 °C).

**Figure 9:**
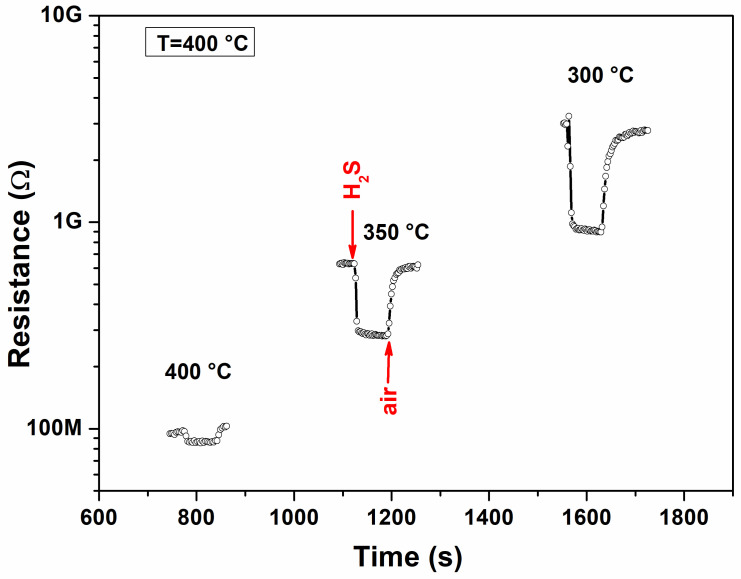
Resistance vs. time of TiO_2_ NPs (400 °C)-based sensor for different operating temperatures (@1.5 ppm H_2_S concentration).

Operating temperature is also an important parameter to take into account for the gas sensing response. Indeed, temperature influences the adsorption/desorption processes of gases occurring on the sensing surface, as well as their reaction rate with adsorbed oxygen on the TiO_2_ surface, and consequently the sensor response. As the above Figure 8 demonstrates, at temperatures lower than 300 °C, the baseline resistance is very high. To evaluate the optimal operating temperature for the sensing tests, the sensor was exposed to 1.5 ppm H_2_S gas at temperatures ranging from 300 to 400 °C (Figure 9). Based on the results obtained, 350 °C appears to be the best operating temperature for this sensor, which displayed a high response to H_2_S and short response/recovery times.

**Figure 10:**
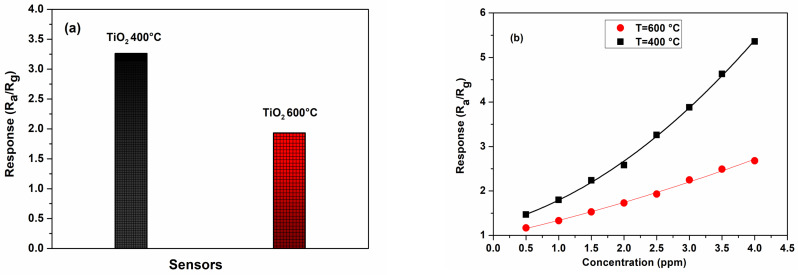
Sensor response of TiO_2_ (400 °C) and TiO_2_ (600 °C) (**a**) at a H_2_S concentration of 2.5 ppm; (**b**) response versus H_2_S concentration at a temperature of 350 °C.

In these operating conditions (Figure 10a), the sensor response of TiO_2_ (400 °C) was registered to be 3.26 for 2.5 ppm of H_2_S, higher than that reported for the TiO_2_ (600 °C) sensor. In addition, we confirmed this result in Figure 10b, which depicts the evolution of the sensor response as a function of the H_2_S concentration for both sensors. This finding is consistent with the results of XRD and SEM analyses. Indeed, it is well-known that when the grain size of the sensing material is small enough, it substantially impacts the gas sensing properties [37,38]. In addition, the sensor annealed at 400 °C has a larger surface-to-volume ratio due to the smaller grain size, thus further justifying the larger response compared to the TiO_2_ (600 °C) sensor. The lower response for the sensor annealed at 600 °C could therefore be related to the improvement in the crystallinity of TiO_2_ nanoparticles. The rearrangement of the atoms is a process that reduces the gas adsorption on the surface [39].

Apart the above structural considerations, the effect of the different phases (anatase and rutile) on the sensing response cannot be excluded. The advantages of using anatase or rutile in gas sensing have been discussed for a long time and depend on many variables such as the target gas and operating temperature. For example, Zakrzewska and Radecka discovered that rutile-dominated TiO_2_ nanomaterials exhibited higher sensitivity towards hydrogen than those with the prevailing anatase [40]. This phenomenon could be accounted for by band alignment and electron transfer from rutile to anatase to facilitate oxygen pre-adsorption. On the contrary, by using density functional theory (DFT) to study the adsorption and reaction of H_2_S on TiO_2_ anatase (101) and rutile (110) surfaces, it has been demonstrated that the adsorption and dissociation of hydrogen sulfide at the TiO_2_ anatase surface require a lower energy barrier compared to at the anatase surface [41]. This latter finding indicates that the presence of anatase at a high concentration (100%) is a factor to take into account when considering the sensor response enhancement of H_2_S.

**Figure 11:**
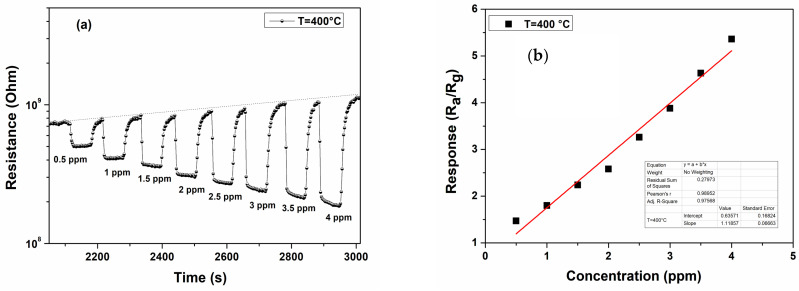
(**a**) Resistance vs. time for different concentrations and (**b**) response vs. concentration of the TiO_2_-NP sensor at an operating temperature of 350 °C.

The sensing performance of the TiO_2_ (400 °C) sensor was further investigated by exposing the fabricated sensors to different concentrations of H_2_S gas. Figure 11a shows the plotted gas response to H_2_S gas sensed by the TiO_2_ (400 °C) sensor at an operating temperature of 350 °C. The response amplitude of the sensor increases with H_2_S concentration in the range of 0.5 to 4 ppm. Moreover, in Figure 11b, it can be observed that the response increases almost linearly with the concentration. The sensor is also sufficiently sensitive at the lowest concentration (0.5 ppm) of H_2_S tested. This result suggests that it can be promising for the sensing of hydrogen sulfide in practical applications.

**Figure 12:**
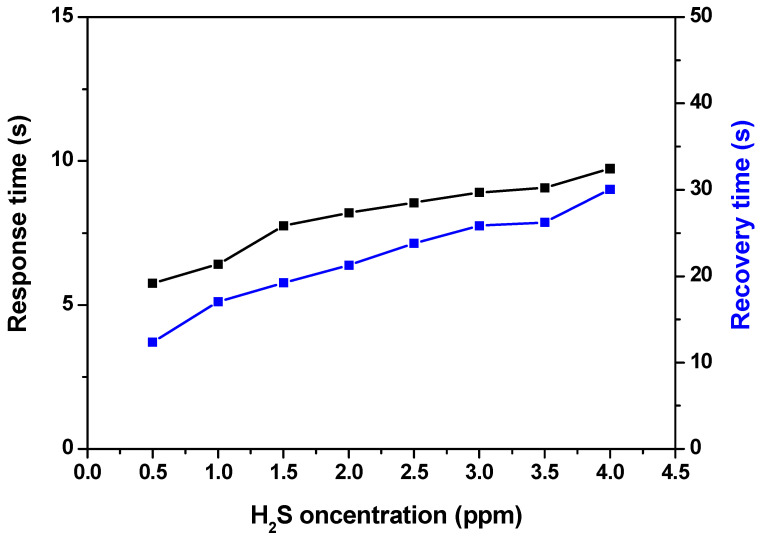
Response and recovery time vs. H_2_S concentrations of TiO_2_-NPs at an operating temperature of 350 °C.

The response and recovery times are two very important characteristics of gas sensors in practical applications. The response and recovery times of the TiO_2_ (400 °C) sensor as a function of various H_2_S concentrations at the operating temperature of 350 °C are presented in Figure 12. The measured response and recovery times are short. Indeed, in the H_2_S concentration range of 0.5 to 4 ppm, the response time is slower than 10 s and the recovery time is slower than 31 s.

**Figure 13:**
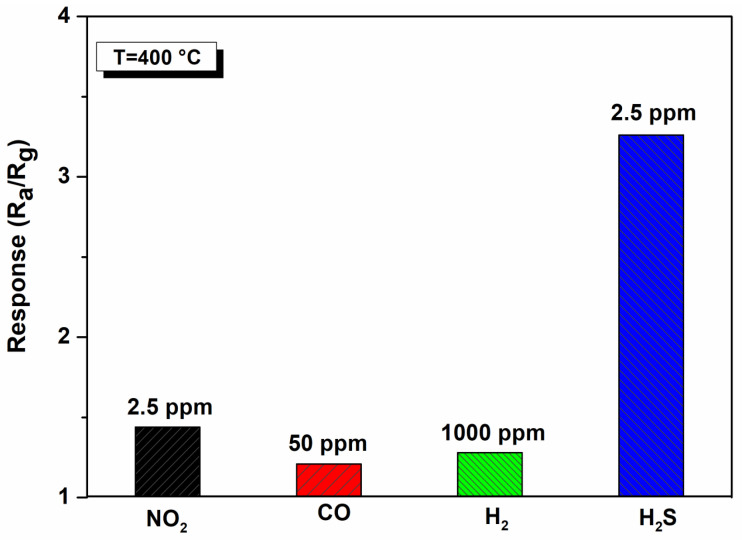
Selectivity pattern of the TiO_2_-NPs sensor at an operating temperature of 350 °C.

The gas sensing selectivity of the TiO_2_ (400 °C) sensor against different gases, i.e., nitrogen dioxide, carbon monoxide, and hydrogen, was also studied (Figure 13). The selectivity patterns indicate that, for all the interfering gases, it presents low responses, and therefore exhibits excellent selectivity to H_2_S.

**Figure 14:**
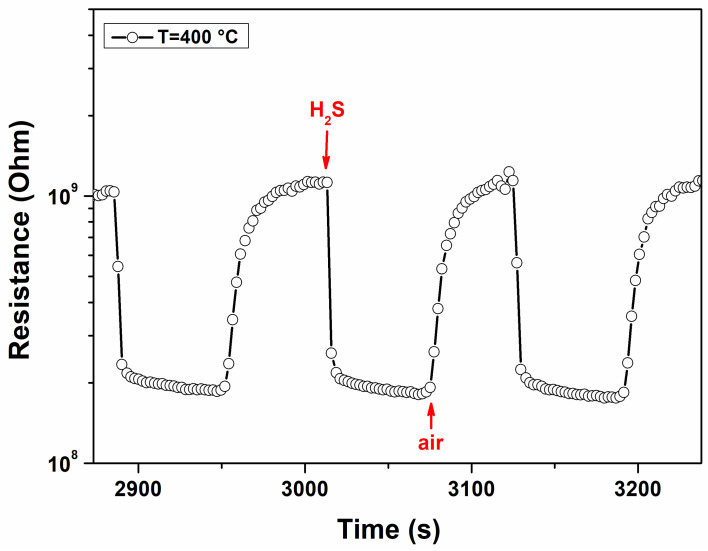
Reproducibility of the sensor response to three pulses of 4 ppm H_2_S in air.

Repeatability is an important indicator for measuring the reliability of the sensor response and the stability of the sensor. Figure 14 shows the reproducibility of the sensor when exposed to three consecutive pulses of 4 ppm of H_2_S gas at the working temperature of 350 °C. It is observed that the response and recovery characteristics are almost reproducible.

### 3.3. Gas Sensing Mechanism

The gas sensing mechanism of the developed sensor is explained by the change in the conductance of the semiconducting TiO_2_ sensing layer. Herein, the conductivity of the sensor is modified by the phenomenon of target gas adsorption-desorption, which causes variations in the electrical conductivity of the sensing layer. The kinetics of gas adsorption and desorption are critical to the performance of gas sensors, influencing their sensitivity, response time, and recovery time. The adsorption process is enhanced by a high surface area, optimal pore size, and high surface energy. Materials such as nanostructured titanium dioxide (TiO_2_) are ideal due to their large surface area and chemical stability, providing numerous active sites for the adsorption of gas molecules. Desorption depends on factors such as binding energy and temperature. Strong interactions between gas molecules and the sensor surface can slow down desorption, resulting in longer recovery times. Increasing the temperature can facilitate faster desorption by providing the necessary energy to overcome binding forces. Sensor design must balance these kinetics to achieve rapid detection and quick recovery. Enhancing selectivity involves modifying the sensor material, such as doping TiO_2_ with elements such as silver or platinum, to tailor the interaction strength with specific gases. These modifications optimize both adsorption and desorption rates, ensuring that the sensor performs reliably and efficiently. Understanding and optimizing these kinetic processes are then essential for developing high-performance gas sensors capable of detecting hazardous gases accurately and swiftly.

When the sensor is exposed to air, oxygen molecules are adsorbed on the surface and extract electrons from the conduction band [39,40]. Oxygen molecules are adsorbed on the active sites of the rough grain surface as $(O_{2}^{-}$, $O^{-}$, and $O^{2-}$) by trapping electrons from the conduction band, which results in an electron depletion region [42,43,44].

**Figure 15:**
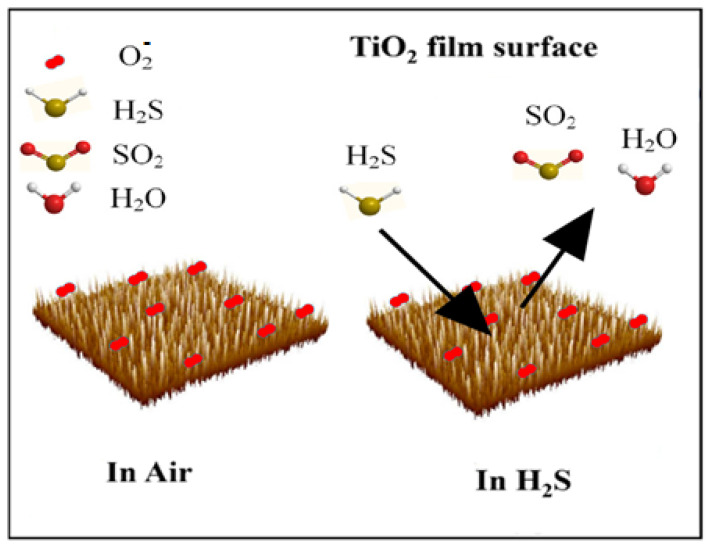
Gas sensing mechanism of TiO_2_ (400 °C) in the presence of H_2_S gas.

When the TiO_2_-based sensor was exposed to H_2_S, it reacted with adsorbed oxygen species and released the trapped electrons back to the TiO_2_ (see Figure 15). Hence, the high sensitivity to hydrogen sulfide can be attributed to its low dissociation energy compared to other gases on TiO_2_ anatase, enabling it to readily react with the adsorbed oxygen [45,46,47,48]) to form SO_2_ and H_2_O, as seen in Equation (3). This causes the bulk release of a large concentration of free electrons, which results in the narrowing of the electron depletion region.

(3) $$2H_{2}S\;\left(g\right)+3O_{2}^{-}\left(ads\right)\to 2H_{2}O\;\left(g\right)+2SO_{2}\left(g\right)+3e^{-}$$

The good performances of our sensor compared to those reported in the literature are reported in Table 3. Remarkably, the sensor response is very high, considering the low H_2_S concentration tested in our case, as well as being faster.

Finally, we planned new tests for the evaluation of further characteristics regarding our sensor. Among these, humidity is well-known to influence the response of resistive sensors. However, the exact behavior is not predictable and various findings have been reported, depending on the metal oxide, the target gas, the operating temperature, and the humidity value [51]. Therefore, tests carried out in different humidity conditions have been planned for the near future.

## 4. Conclusions

In summary, titanium dioxide nanoparticles were prepared by a modified sol-gel technique and then annealed at different air temperatures (400 °C and 600 °C). The synthesized samples exhibit a polycrystalline structure, characterized by grains of pure anatase on TiO_2_ (400 °C) and with about 25% of rutile in TiO_2_ (600 °C). Subsequently, the samples were used to fabricate gas sensors for H_2_S. The TiO_2_ (400 °C)-based gas sensor was found to display the best performances in terms of high response, fast response/recovery, and good selectivity when operating at 350 °C. The obtained results confirm that the TiO_2_ sample treated at 400 °C can be considered very promising for the detection of low H_2_S concentrations and suitable for a variety of environmental applications.

## Figures and Tables

**Table 1:** Fitting parameters of the Rietveld refinement on DRX profiles of TiO_2_ annealed at 400 °C and 600 °C.

Sample	χ^2^	Bragg R-Factor	R Factors	Rp	Rwp	Rexp
TiO_2_ (400 °C)	1.86	11.0	11.2	5.34	6.47	4.75
TiO_2_ (600 °C)	2.57	2.83	1.55	4.37	5.37	3.35

**Table 2:** Crystallographic properties of TiO_2_ annealed at 400 °C and 600 °C.

Sample	Crystalline Phase	Space Group	Lattice Parameters (Å)	Crystallite Size (nm)	Strain (×10^−5^)	Phase Composition (%)
TiO_2_ (400 °C)	Tetragonal (anatase)	I4_1_/amd	a = b = 3.7761 c = 9.4950	11	0.00359	100
TiO_2_ (600 °C)	Tetragonal (anatase)	I4_1_/amd	a = b = 3.7868 c = 9.5174	51	0.00224	76.95
	Orthorhombic (rutile)	Pnma	a = 5.1124 b = 3.2054 c = 6.0871	-	-	23.05

**Table 3:** Comparison of the sensing performances of the TiO_2_-based sensor with other sensors reported in the literature.

Material	H_2_S (ppm)	Response (Ra/Rg)	Temperature (°C)	Response Time (s)	Recovery Time (s)	Reference
TiO_2_ nanoplates (Anatase)	10	4.8	300	10	-	[46]
TiO_2_ nanotube (Anatase)	50	26	300	22	6	[6]
TiO_2_ nanowires (Rutile)	80	11	140	-	-	[47]
TiO_2_-Al_2_O_3_ (Rutile)	1000	38.7	650	390	480	[49]
Ag-doped TiO_2_ nanofiber	100	8.5	350	-	-	[44]
CuO doped TiO_2_ nanoparticle (Anatase)	50	1.78	Room temperature	14	22	[50]
TiO_2_ nanoparticles (Anatase)	2.5	3.3	350	8	23	This work

## Data Availability

The original contributions presented in the study are included in the article, further inquiries can be directed to the corresponding author.
